# Cost of simulation-based mastery learning for abdominal ultrasound

**DOI:** 10.1186/s12909-023-04919-5

**Published:** 2023-12-05

**Authors:** Julie H. Post, Kristina E. Teslak, Martin G. Tolsgaard, Sten Rasmussen, Mikkel L. Friis

**Affiliations:** 1 Aalborg University Hospital, Aalborg, Denmark; 2 Copenhagen Academy for Medical Education and Simulation, Aalborg, Denmark; 3 NordSim, Center for Skills training and Simulation, Aalborg University Hospital, Aalborg, Denmark

**Keywords:** Radiology, Simulation-based medical education, Learning curves, Virtual reality, Ultrasound training, Ultrasound assessment, Simulation-based ultrasound training, Mastery learning, Ultrasound simulation

## Abstract

**Background:**

Ultrasound is an essential diagnostic examination used in several medical specialties. However, the quality of ultrasound examinations is dependent on mastery of certain skills, which may be difficult and costly to attain in the clinical setting. This study aimed to explore mastery learning for trainees practicing general abdominal ultrasound using a virtual reality simulator and to evaluate the associated cost per student achieving the mastery learning level.

**Methods:**

Trainees were instructed to train on a virtual reality ultrasound simulator until the attainment of a mastery learning level was established in a previous study. Automated simulator scores were used to track performances during each round of training, and these scores were recorded to determine learning curves. Finally, the costs of the training were evaluated using a micro-costing procedure.

**Results:**

Twenty-one out of the 24 trainees managed to attain the predefined mastery level two times consecutively. The trainees completed their training with a median of 2h38min (range: 1h20min-4h30min) using a median of 7 attempts (range: 3–11 attempts) at the simulator test. The cost of training one trainee to the mastery level was estimated to be USD 638.

**Conclusion:**

Complete trainees can obtain mastery learning levels in general abdominal ultrasound examinations within 3 hours of training in the simulated setting and at an average cost of USD 638 per trainee. Future studies are needed to explore how the cost of simulation-based training is best balanced against the costs of clinical training.

**Supplementary Information:**

The online version contains supplementary material available at 10.1186/s12909-023-04919-5.

## Background

Ultrasound is increasingly used for point of care (POC) examinations in several medical and surgical specialties [1]. Although ultrasound-based diagnoses can be made using POC ultrasound, it is highly operator-dependent [2–4]. To ensure adequate standards of practice, the international ultrasound societies recommend a certain volume of examinations to be completed under supervision before independent practice [2]. However, trainees attain ultrasound skills at different rates. Some trainees may not achieve adequate performance levels within the planned number of cases, whereas other trainees require far less training than the required number of cases to achieve competency. This challenges current time- and volume-based learning approaches recommended by the international ultrasound societies [5].

An alternative approach is competency-based training until trainees reach a predefined mastery level. This type of training is called mastery learning and ensures a uniform educational outcome among all trainees [6]. Mastery learning may require large resources in the clinical setting because it demands unlimited supervision and assessments by ultrasound experts [5–10]. Using simulation-based ultrasound training may lower the need for clinical supervision because new trainees can be trained to a certain competency-level in the simulated setting with automated feedback and little involvement from clinician experts. The automated feedback is provided by the simulator in terms of pass/fail decisions on technical and diagnostic performances [8–10]. Ultrasound simulators and their use are expensive, and few studies exist to determine the costs of mastery learning levels in the simulated setting [7]. This gap in our current knowledge is important to fill in order to provide high-value low-cost education in the future – in particular in the context of ultrasound training, in which there are few patient risks associated with supervised clinical practice.

In this study, we aimed to explore mastery learning curves for general abdominal ultrasound in the simulated setting and to determine its associated costs.

## Methods

### Setting and design

**Fig. 1:**
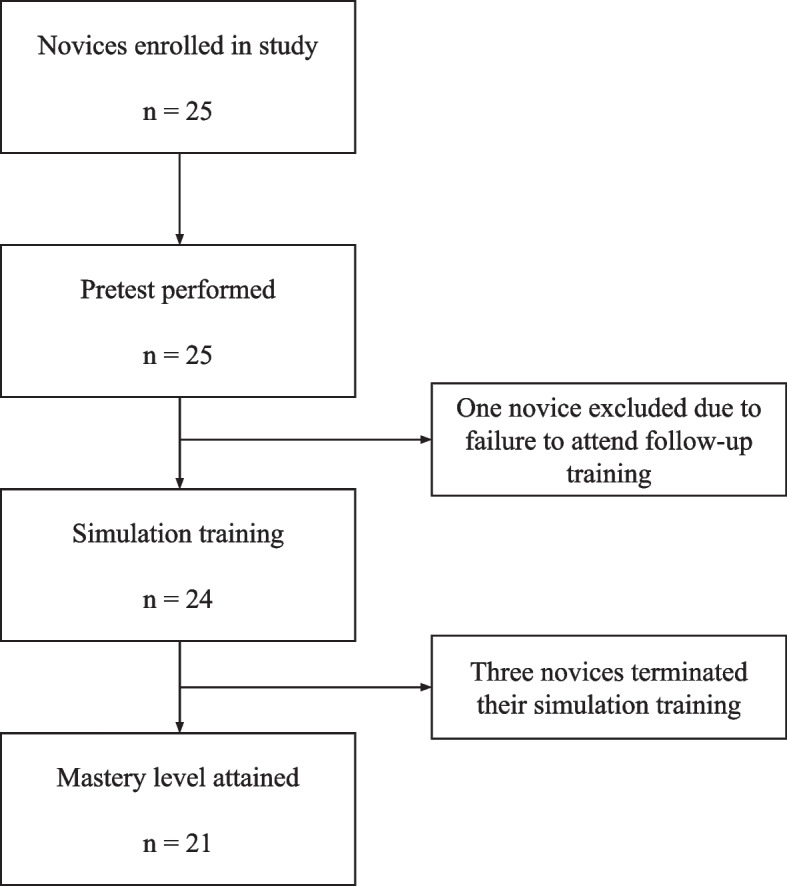
Flow chart depicting the study set-up

The present study is based on a previous validation study (unpublished data), in which we collected validity evidence for simulation-based assessments of general abdominal ultrasound skills (Fig. 1). All assessments and training were conducted using a virtual reality simulator (ScanTrainer®, MedaPhor, Cardiff, UK) that provides automated feedback (metrics) after completion of each training module. In our study, we used a training program consisting of five modules reflecting examination of the liver, gallbladder, pancreas, kidneys, and spleen. After completing a round of the training program, a test score was calculated based on feedback metrics, measured as a percent of the maximum score.

To conduct this study, the contrasting groups method was applied [11]. We included a group of ultrasound trainees consisting of medical students and an expert group of radiologists from Aalborg University Hospital. The experts were required to complete the entire test once to establish a mastery learning level. This mastery learning level was used as a benchmark to guide when the trainees had attained competency in the simulated setting. The medical students were invited to continue their training until the pre-defined mastery level was attained twice in a row. This criterion was introduced to reduce the chance of passing by coincidence and because expertise is characterized by consistency in performance. The study was registered by Danish Data Protection (Protocol no. 2021–106) of Region North Jutland, Denmark.

### Participant recruitment

The participants were third-year medical students from Aalborg University in Denmark. All participants had been taught basic abdominal anatomy and had no previous experience with ultrasound or ultrasound simulation.

### Equipment

This study was conducted on a virtual reality transabdominal ultrasound simulator, ScanTrainer® (MedaPhor, Intelligent Ultrasound). The simulator provides haptic feedback, data from real patient scans, and features of actual ultrasound machines, all of which contribute to simulator fidelity. The study involved five modules for upper abdominal ultrasound including various tasks relevant for the examination of the liver, spleen, kidney, pancreas, and gallbladder.

### Learning curves

The participants all received a standardized introduction and warm-up exercises. During all training sessions, participants practice independently on the simulator. The only feedback provided was from the automated simulator metrics. A simulator instructor was available in case of technical errors or questions but was not allowed to provide feedback to the participants. The simulator instructor ensured data integrity and completeness during all training. A single training round consisted of a total of five modules identified in a previous validation study [unpublished data]. After each round of training, a total score was calculated as the percent of the maximum score based on simulator metrics from each of the five modules. To attain the mastery learning level 31.5 out of 37 (85.14%) simulator metrics needed to be passed.

### Statistical analysis

The learning curves of the participants attempting to reach mastery level were determined by built-in automatic simulator metrics. Failed metrics were provided a score of 0 and passed metrics were assigned a score of 1. The total maximum score (37) was calculated into the percent of the maximum score (0–37). The learning curve was demonstrated as the progression in mean simulator scores for each round of training. The last two attempts of the trainees to attain mastery level were compared to the pretest of the experts by a Mann-Whitney U test, and a Kaplan-Meier plot was used to illustrate the time spent to reach mastery level.

### Economic evaluations

To evaluate the costs of creating the program and the trainees to achieve mastery level, Levine’s ‘Ingredients Method’ was used [12]. The method includes four steps, 1) specification of resources used (for example time, materials, equipment), 2) determination of the quantities (unit) of each resource, 3) determination of the unit price of each resource, and 4) multiplication of the units and price resulting in determination of total cost per resource [12]. Equipment cost (i.e. simulator cost) was depreciated over a five-year period estimating an average use of 50 days per year.

All costs were determined based on existing market prices. For example, salaries were identified based on existing labour agreements. For the trainees, we calculated their time as opportunity costs, that is, the foregone benefit of an option not pursued or chosen (for example, working as a student assistant instead of participating in a research study or using facilities for training in our study instead of renting them out). The calculations of cost were based on DKK and were converted into USD with an exchange rate of 748.75 [13]. Finally, a sensitivity analysis was performed to account for the diminishing costs per trainee with an increased volume of trainees. This allowed us to consider program development and test validation costs into the cost of mastery learning for different scenarios (for instance, a hospital unit with very few trainees or a large simulation center with a large number of trainees).

## Results

**Fig. 2:**
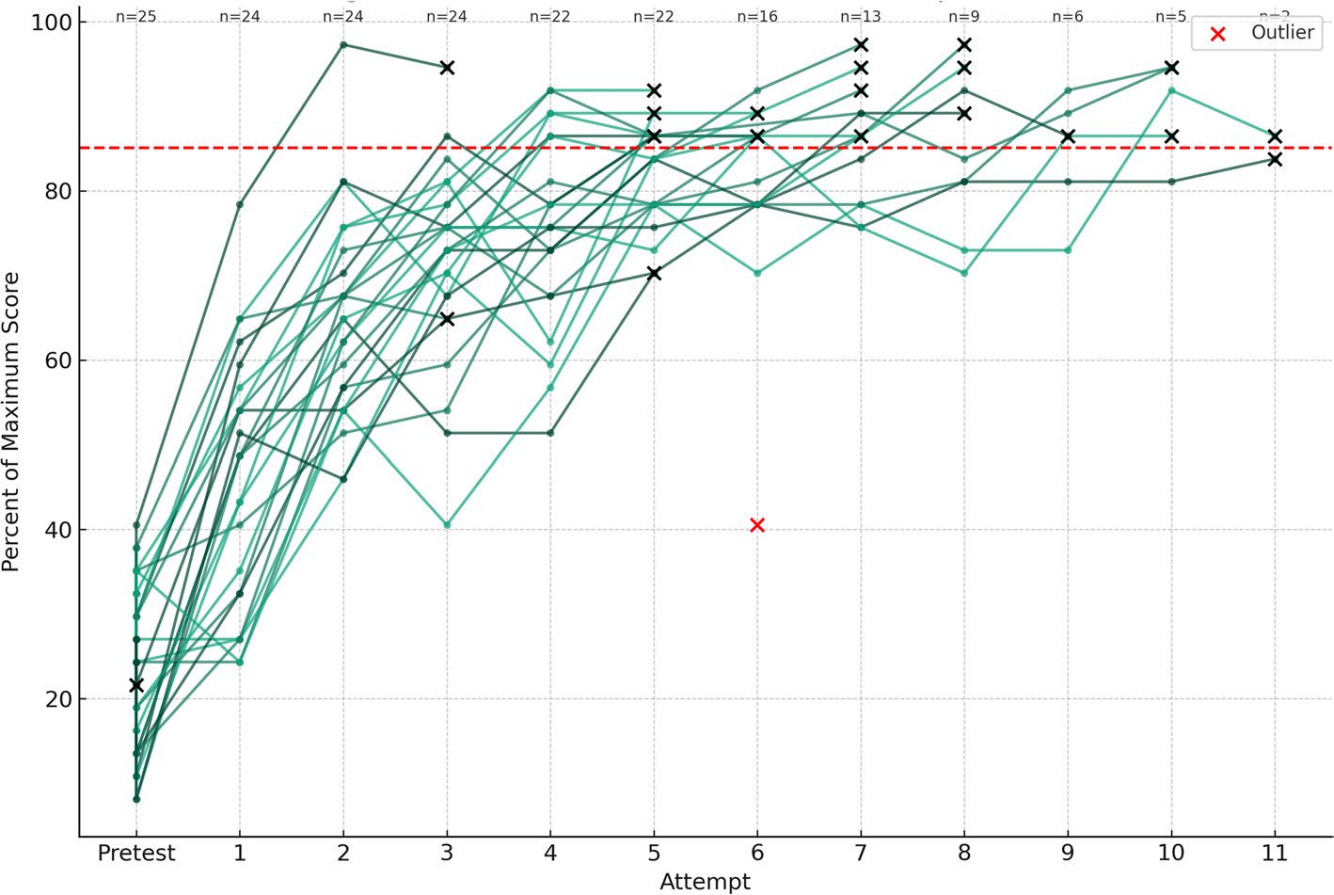
Learning curves with individual participant scores. A horizontal line marks the mastery learning level at 85%. We added small amounts of noise to each participant on the x-axis to allow better discrimination between each participant. Participants practiced until achieving the mastery learning level twice and then dropped out (number of participants shown in the top row)

The median sum score of the experts constituted the mastery learning level to be 85% of the maximum score. A total of 25 trainees met the inclusion criterion and were enrolled, and after the initial pretest 24 trainees proceeded with simulation training and thereby attempted to reach the defined mastery learning level [unpublished data]. Baseline characteristics are shown in Table 1. Of the 24 trainees, 21 (88%) attained the mastery learning level achieving a minimum sum score of 85% twice. One trainee was excluded after completing the pretest from the study by since the participant was unable to follow the subsequent training. Additional three trainees were unable to attain mastery learning level within the designated time frame of 4 weeks. Their median number of attempts and training time in minutes was approximating the same distribution as the trainees attaining mastery learning level (5 attempts, 2h8min). To attain a mastery learning level, participants spent a median of 2h38min (range 1 h 20 min- 4 h 30 min) using a median of seven attempts on the simulator (range 3–11 attempts). Learning curves for trainees are illustrated in Fig. 2 showing a correlation between learning plateau and mastery learning level.

**Table 1:** Baseline demographics of the participants

Characteristics	Trainees (*n* = 24)	Attained mastery learning level (*n* = 21)	Failure to attain mastery learning level(*n* = 3)
Median age (range)	23 (21–28)	23,5 (21–28)	23 (22–24)
Women, n	12	9	3
Men, n	12	12	0
Median years of experience	0	0	0

**Fig. 3:**
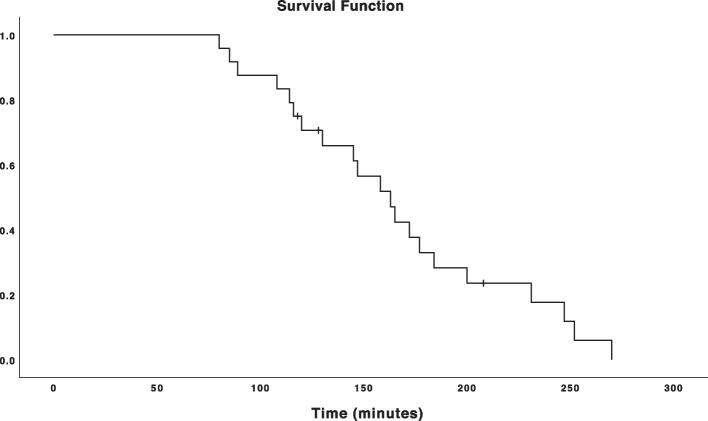
Kaplan-Meier plot. The time spent on the ScanTrainer for trainees before attaining mastery learning level is demonstrated. Trainees that did not manage to attain a mastery learning level are displayed as censored. The time needed to reach mastery learning level ranged from 1h20min to 4h30min with a median of 2h38min

The Kaplan-Meier plot below (Fig. 3) depicts the time spent by the trainees to attain a mastery learning level. Three trainees (12.5%) did not attain a mastery learning level, which is marked in Fig. 3 as censored observations.

Two Mann-Whitney U tests were performed to compare the performance level of the experts and the last two attempts of the trainees; one test only included the trainees attaining mastery learning level and another test included all trainees; *p* = 0.007 (CI: 21–36, SD: 2.47) and *p* = 0.049 (CI: 19–36, SD 3.39), respectively.

### Evaluation of costs

To assess the costs of training of trainees to attain a mastery learning level, the resources used in the process were specified and calculated. The costs of providing validity evidence for the automated assessment scores used for all training and feedback purposes in this study are presented in Table 2. The ultrasound simulator cost was USD 42,204 and assuming depreciation over 5 years and 50 days of use per year, the hourly rate was determined at USD 23. Based on the micro-costing procedure, the direct costs of simulation-based mastery learning were calculated to be USD 639 per trainee passing the expert criterion, as shown in Table 3.

**Table 2:** Indirect cost of developing the simulation program and for examining the validity of assessments used for subsequent mastery learning training. All costs are in USD

Expense	Expense per hour (USD)	Quantity	Total (USD)
**Program development costs (administration)**	42.60	1	42.60
**Training simulator instructors**	21.37	16	341.92
**Simulator instructor**	21.37	28	598.36
**Specialists in radiology**	64.51	3	193.53
**Trainee (opportunity cost)**	35.39	25	884.75
**Depreciation of simulator**	22.81	28	638.68
**Facility costs**	26.71	45	1201.95
**Total**			**3901.79**

**Table 3:** Direct cost of simulation-based mastery learning of a single trainee without taking development cost into account. Quantities are based on the mean training time found in this study. All costs in USD

Expense	Expense per hour (USD)	Quantity	Total (USD)
**Simulator instructor**	21.37	6	128.22
**Trainees (opportunity costs)**	35.39	6	212.34
**Depreciation of simulator**	22.81	6	136.86
**Facility costs**	26.71	6	160.26
**Total**			**638**

A sensitivity analysis was performed to account for the reduced impact of program development and test validation costs with 1, 10, 50, 100, 200 and 500 participants, which can be described by the following equation y = (637.68x + 3901.79)/x, where y is cost per trainee and x is the number of trainees, Table 4. To allow modeling of our data, we have made an Excel file available in the Supplementary materials that can be used for repeating sensitivity analyses by inputting costs relevant to other institutions and contexts.

**Table 4:** The cost per trainee is dependent on the total number of trainees participating in the mastery learning program. With more than 50 trainees, the program development and test validation cost become less than about 10% of the total costs. All costs are in USD

Number of trainees	Total price (USD)	Price pr trainee (USD)
1	4539.47	4541
10	10,278.68.6	1029
50	35,786,786	719
100	67,760	678
200	131,438	659
500	322,740	647

## Discussion

For complete ultrasound novices, mastery learning levels in general abdominal ultrasound can be attained within 6 hours of simulation-based practice in our study. Compared with the large volume of scans before independent practice recommended by the international ultrasound societies, the learning curves presented in our study may seem very steep. This is only partially true, as the learning curves demonstrated in the simulated setting do not translate to directly clinical performances. For example, a study of gynecological ultrasound training demonstrated that trainees who completed simulation-based training before clinical practice still required high levels of supervision from senior colleagues over their first 6 months of training. However, the amount of supervision needed was substantially less and the quality of scans produced was far better than for trainees, who did not complete any simulation-based training prior to their clinical training [10, 14]. Compared to these prior studies, our current work differs in several respects.

Previous studies focusing on simulation-based ultrasound training have reported significantly shorter learning curves before attaining mastery learning levels – for example, between three to 4 hours for simulation-based obstetric [15], gynecological ultrasound [16], or FAST examinations [5]. Our training program focused on the skills needed for a diagnostic abdominal ultrasound, which may be more comprehensive than the knowledge and skills needed for procedures such as gynecological ultrasound or the FAST examination. The level of diagnostic skills needed also seems to differ for diagnostic examinations compared to point-of-care examinations, which may explain the longer learning curves.

There are few studies on simulation-based ultrasound training that have involved cost. In one previous study, the authors linked transvaginal ultrasound training costs to reductions in patient waiting time [8]. The cost of training in that study was USD 445 (EUR 448) per participant for training on two different types of simulators but for a much simpler procedure (transvaginal cervical scans), which is less than the expenditure in our study [8]. The differences are driven by different estimates of curriculum development costs and equipment depreciation costs [8]. For these reasons, we have conducted a sensitivity analysis in our study to account for the impact of having different volumes of trainees on the incremental cost per additional new student. This step is in accordance with best current practices for reporting economic evaluations in health professions education [12] and highlights how vulnerable cost evaluations can be if not accounting for different scenarios through sensitivity analyses.

Sensitivity analyses can be applied for a number of other drivers of overall cost such as instructor salaries or opportunity costs (the potential benefits missed when choosing one alternative over another) for trainees. These expenditures may differ widely across settings, for example, a simulator instructor cost USD 23 per hour in our unit but can likely cost less or significantly more in other settings. These assumptions can be modeled using the Sensitivity Analysis file in the Supplementary materials. Regardless of differences between institutions and settings, it remains important to acknowledge the role of opportunity costs. Although the participants in our study were volunteers who were not paid, their time cannot be considered to have no economic value. If the participants had not spent their time on our study, they may have used it elsewhere and in an open market, they would have been able to work for a given cost. Opportunity cost in this study is estimated based on agreed salary agreements. If we had falsely reported this opportunity cost as zero, we may have vastly underestimated the true costs of simulation-based ultrasound training. Our study is to the best of our knowledge the first to report the cost of simulation-based mastery learning using best practices such as micro-costing procedures for cost estimation and sensitivity analysis for evaluating different underlying assumptions and drivers of cost.

In the context of ultrasound training, an increasing body of literature is supporting the effectiveness of simulation-based training [17, 18]. We still have limited knowledge on how to use and prioritize the different available training opportunities and resources best, such as supervised clinical practice, simulation, online learning etc. For invasive procedures that involve some level of patient risk, such as laparoscopy, it is crucial that trainees are not allowed to practice with real patients before mastery of basic skills. This makes simulation-based training an ethical imperative [19]. In the case of ultrasound, there are few known patient risks associated with supervised clinical practice, even for novice trainees. This makes the study of cost in relation to simulation-based ultrasound training an important subject for future research along with studies examining the impact of simulation-based ultrasound training on patient care and outcomes [10].

There are a number of limitations in our study. Our study is also limited by selection bias in the population of trainees, we recruited; a well-established transfer gap to clinical performances; and considerable ceiling effect from teaching the test a repeated number of times. Furthermore, it was a single-centre study, which limits the generalizability of the cost estimates. We handled this limitation through sensitivity analysis to account for the fact that costs differ depending on context. The cost evaluations included a number of assumptions that were made and reported according to existing guidelines on how to conduct cost and value studies in health professions education. Whereas some of these cost estimates were straight forward, such as hour rates for simulator instructors, while others were not, such as depreciation of simulator equipment. These limitations are methodological and call for increased attention to how we standardize future descriptions and evaluations of costs in future studies.

## Conclusion

Complete trainees can obtain mastery learning levels in general abdominal ultrasound examinations within 3 hours of training in the simulated setting and at an average direct cost of USD 638 per trainee and with total costs highly dependent on the volume of trainees. Future studies are needed to explore how the cost of simulation-based training is best balanced against costs of clinical training.

### Supplementary Information

**Additional file 1.**

**Additional file 2.**

**Additional file 3.**

## Data Availability

All data generated and analysed during this study are included in this published article. Raw data of the study regarding baseline demographics, metric scores and rounds and time for reaching mastery level is found as supplementary material, together with data to conduct a sensitivity analysis.
